# Long Non-Coding RNA–Derived Peptides as a Novel Source of Tumor Neoantigens: Expanding the Immunopeptidome Beyond Canonical Coding Regions

**DOI:** 10.3390/biology15070538

**Published:** 2026-03-27

**Authors:** Ismael López-Calvo, Inés Bao-Camacho, Samuel Martín-Revuelta, Cora Rey-Souto, Anahir Franco-Gacio, José Manuel Pérez-Martínez, Iván Sandino-Somoza, Álvaro Mourenza, Esther Rodríguez-Belmonte, Mónica Lamas-Maceiras, M Esperanza Cerdán, Aida Barreiro-Alonso, Ángel Vizoso-Vázquez

**Affiliations:** 1 Centro Interdisciplinar de Química e Bioloxía (CICA), Universidade da Coruña, Campus de Elviña, As Carballeiras, s/n, 15071 A Coruña, Spain; ismael.lopez1@udc.es (I.L.-C.); ines.bao.camacho@udc.es (I.B.-C.); s.mrevuelta@udc.es (S.M.-R.); cora.rey.souto@udc.es (C.R.-S.); anahir.franco@udc.es (A.F.-G.); jose.manuel.perez@udc.es (J.M.P.-M.); ivan.sandino@udc.es (I.S.-S.); alvaro.mourenza@udc.es (Á.M.); monica.lamas@udc.es (M.L.-M.); esper.cerdan@udc.es (M.E.C.); 2 Instituto de Investigación Biomédica de A Coruña (INIBIC), As Xubias de Arriba 84, 15006 A Coruña, Spain; esther.belmonte@udc.es; 3 Facultade de Ciencias, Universidade da Coruña, Campus de A Zapateira, A Fraga, s/n, 15071 A Coruña, Spain; 4 CITIC-Research Center of Information and Communication Technologies, Universidade da Coruna, 15071 A Coruña, Spain

**Keywords:** lncPEP, smORFs, MHC-I, dark proteome, proteogenomics, immunotherapy, precision oncology

## Abstract

Cancer immunotherapy has transformed the clinical management of several malignancies; however, its efficacy remains limited in tumors with low mutational burden and restricted availability of classical mutation-derived neoantigens. In this context, increasing evidence indicates that the tumor immunopeptidome extends far beyond canonical protein-coding regions, incorporating peptides derived from non-coding transcripts through non-canonical translation mechanisms. Long non-coding RNAs (lncRNAs), traditionally regarded as transcriptional or post-transcriptional regulators, have recently emerged as an unexpected source of small open reading frame-encoded peptides (lncPEPs). A subset of these peptides is processed and presented by major histocompatibility complex class I molecules, generating tumor-specific neoantigens capable of eliciting CD8^+^ T cell responses. Owing to the high tissue and context specificity of lncRNA expression, lncRNA-derived neoantigens offer unique advantages over mutation-based targets, including increased tumor selectivity and potential recurrence across patient subsets. In this review, we synthesize current knowledge on the biogenesis, detection, and immunogenic potential of lncRNA-derived peptides, highlighting experimental and computational strategies for their identification within the cancer immunopeptidome. We discuss the challenges associated with their validation and clinical translation, as well as their relevance for the development of vaccines and adoptive T cell–based therapies. Finally, we illustrate these concepts using epithelial ovarian cancer as a representative model of low-mutational-burden tumors, where lncRNA-derived neoantigens may help overcome current limitations of immunotherapy and enable patient stratification for personalized treatment approaches.

## Simple Summary

Cancer immunotherapy has significantly improved the clinical management of several malignancies, yet its efficacy remains limited in tumors with low mutational burden, where the availability of classical mutation-derived neoantigens is restricted. Recent advances in proteogenomics and immunopeptidomics indicate that the tumor immunopeptidome extends beyond canonical protein-coding regions and includes peptides derived from non-coding transcripts through non-canonical translation mechanisms. In this review, we synthesize current evidence on long non-coding RNA (lncRNA)–derived peptides (lncPEPs) as an emerging source of tumor neoantigens. We summarize the molecular mechanisms underlying their biogenesis, including the translation of small open reading frames within lncRNAs, and discuss experimental and computational strategies used for their identification, such as translatomics, proteogenomics, and immunopeptidomics. We also examine approaches for validating their immunogenicity and assessing their potential as therapeutic targets. Finally, we highlight epithelial ovarian cancer as a representative model of low-mutational-burden tumors in which lncRNA-derived neoantigens may expand the repertoire of actionable antigens. Collectively, these findings suggest that lncRNA-derived peptides represent a promising and largely unexplored class of targets that could broaden the landscape of cancer immunotherapy and support the development of personalized therapeutic strategies.

## 1. Introduction

Traditional cancer treatment typically follows a sequential approach involving surgical resection of the tumor, preceded or followed by chemotherapy and/or radiotherapy. However, conventional therapies face significant limitations, including the development of drug resistance, which renders treatments ineffective over time, and cause high recurrence rates, particularly in aggressive malignancies such as ovarian, pancreatic, and glioblastoma cancers. Furthermore, many cancers are diagnosed at advanced stages, when conventional treatments are substantially less effective [1]. In this context, there is an urgent need to identify alternative therapeutic strategies that enable earlier diagnosis and more effective treatment of advanced disease, ultimately improving patient outcomes.

One of the most promising strategies is cancer immunotherapy, which aims to enhance the antitumor immune response against specific tumor antigens. Among the most prominent approaches are personalized cancer vaccines that stimulate the patient’s immune response [2,3]; chimeric antigen receptor T-cell therapy (CAR-T therapy) [4,5], which involves genetically modifying the patient’s own cells to recognize specific antigens; and targeted antibody therapies designed to eliminate tumor cells expressing particular antigens [6]. In addition, immune checkpoint blockade therapies targeting inhibitory molecules such as cytotoxic T-lymphocyte antigen-4 (CTLA-4) and programmed cell death protein-1 (PD-1) have demonstrated considerable clinical benefit in multiple tumor types.

Within this framework, immune recognition is largely determined by the immunopeptidome, defined as the complete repertoire of peptides presented on major histocompatibility complex (MHC) molecules at the cell surface. Antigen presentation through MHC molecules mediates immune surveillance and antitumor responses by enabling T lymphocyte recognition of malignant cells. Importantly, the immunopeptidome is a dynamic system that continuously adapts in response to tumor-intrinsic alterations and microenvironmental stimuli [7]. Identifying novel antigens that can serve both as biomarkers and therapeutic targets has therefore become a major objective in cancer research. This has led to increasing interest in neoantigens, peptides generated as a consequence of tumor-specific molecular alterations, including genomic mutations, post-transcriptional events, and dysregulated RNA processing [8,9].

Traditionally, most efforts to identify tumor neoantigens have focused on mutations occurring within protein-coding regions of the genome. However, recent advances in proteogenomics and immunopeptidomics have demonstrated that a substantial fraction of the tumor antigen repertoire originates from non-canonical genomic and transcriptomic regions, revealing an expanded landscape of potential antigenic sources.

Cancer is characterized by widespread transcriptional dysregulation in which RNA molecules play a central role. Advances in high-throughput sequencing technologies have enabled the identification of numerous non-coding RNAs (ncRNAs), transcripts that were previously considered non-functional but are now recognized as key regulators of physiological and pathological processes, including tumorigenesis [9].

Long non-coding RNAs (lncRNAs), defined as transcripts longer than 200 nucleotides with limited or non-canonical protein-coding potential, have been implicated in diverse cellular functions such as chromatin remodeling, transcriptional and translational regulation, inflammation, cell differentiation, and cancer progression through modulation of gene expression [9,10]. More recently, accumulating evidence has demonstrated that a subset of lncRNAs contains small open reading frames (smORFs) capable of producing short peptides, commonly referred to as lncRNA-derived peptides (lncPEPs). These peptides can participate in tumor-related processes and, in some cases, contribute to the repertoire of tumor neoantigens presented by MHC class I (MHC-I) molecules [11].

The generation of antigenic peptides from lncRNAs involves non-canonical translation mechanisms and subsequent antigen processing pathways that ultimately enable their presentation to cytotoxic T lymphocytes, linking RNA biology with adaptive immune recognition. Importantly, the emergence of lncPEPs expands the conceptual boundaries of the cancer immunopeptidome beyond canonical protein-coding regions, revealing a previously underappreciated layer of antigenic diversity. Owing to their often tissue- and context-specific expression patterns, lncRNA-derived peptides may represent promising candidates for tumor-specific immune targeting, particularly in malignancies with low mutational burden where conventional mutation-derived neoantigens are relatively scarce. A comparison between mutation-derived and lncRNA-derived neoantigens is summarized in Table 1.

Collectively, this evidence highlights lncRNA-derived neoantigens (neoAgs-lncRNA) as a largely unexplored but potentially transformative class of targets for cancer immunotherapy and precision oncology. In this review, we synthesize current knowledge on the biogenesis, antigen presentation, identification strategies, and functional validation of lncPEPs, with a specific focus on their emerging role as a distinct source of tumor neoantigens. While previous reviews have broadly addressed non-canonical antigen sources and the dark proteome, this work specifically examines lncPEPs and the mechanisms underlying their generation and immune recognition. We also discuss their translational potential for the development of cancer vaccines and adoptive T cell–based therapies, illustrating these concepts using epithelial ovarian cancer as a representative model of low-mutational-burden tumor. Given the emerging and interdisciplinary nature of this field, the present work follows a narrative synthesis approach integrating mechanistic, technological, and translational perspectives.

## 2. Tumor Neoantigens: Beyond Coding Mutations

### 2.1. Classical Definition of Tumor Neoantigens

Nowadays, tumor antigens can be divided into three broad categories: tumor associated antigens (TAAs), tumor specific antigens (TSAs), and germline/cancer testis antigens (CTAs) [12,13]. Neoantigens, the main group of TSAs, differ from tumor-associated antigens by their exclusive presence in tumor tissue as they are generated as a consequence of molecular alterations that are unique to tumor cells. On the other hand, TAAs are non-mutated self-antigens and although rare, in some cases they can be expressed in both cancerous and healthy cells. Because neoantigens are perceived as non-self, they trigger immune responses that escape both central and peripheral tolerance and this makes them highly specific targets for immunotherapy [8,14,15]. Neoantigens can be categorized based on their distribution: shared/public neoantigens that occur across different tumor types or in multiple patients [16,17,18,19], versus private/personalized neoantigens that are unique to specific tumors or individuals.

Traditionally, neoantigens were defined as peptides generated by tumor cells as a consequence of somatic mutations in protein-coding genes. However, advances in proteogenomics and immunopeptidomics have expanded this definition [20]. Neoantigens are now understood to originate not only from somatic mutations, but also from alternative reading frames and non-coding transcripts [21]. A study carried out by Laumont et al. in murine cell lines and in B-lineage acute lymphoblastic leukemia and lung cancer patient samples identified 40 TSAs, of which 90% derived from non-coding regions [22]. These findings highlight the existence of an expanded tumor antigen repertoire beyond canonical coding regions, in which non-coding transcripts, including lncRNAs, represent an emerging source of cryptic peptides that can contribute to the tumor immunopeptidome. Importantly, only a subset of these peptides may ultimately function as immunogenic neoantigens capable of eliciting T-cell responses. Together, these observations suggest that non-canonical translation products derived from lncRNAs may expand the repertoire of potential tumor antigens beyond classical mutation-derived neoantigens. This observation supports the growing evidence that non-canonical translation events may contribute substantially to the diversity of the tumor immunopeptidome.

The clinical relevance of neoantigens lies in their recognition as foreign by the immune system, triggering an antitumor response mediated by CD8^+^ T lymphocytes. Moreover, their presence has been associated with improved patient prognosis [8]. The identification of shared neoantigens would enable their use in personalized immunotherapies, such as cancer vaccines or T cell receptor-based treatments, representing a major milestone in oncology, particularly in tumors with a low mutational burden, such as esophageal cancer, where mutation-derived neoantigens are scarce [21]. Therefore, alternative sources of neoantigens are of particular interest in tumor types where the mutational landscape alone does not provide sufficient immunogenic targets.

### 2.2. Non-Canonical Sources of Neoantigens

As tumor neoantigens are not limited to epitopes originating from non-synonymous single-nucleotide variants in the coding exome, in the following subsection, we will examine mechanisms underlying non-canonical neoantigen generation that contribute to the expansion of the cancer immunopeptidome.

#### 2.2.1. Alternative Reading Frames

Frameshift mutations can generate new peptides, neo-open reading frame peptides (NOPs), found in tumors and show enrichment within cancer driver genes [23]. Frameshift mutations can be associated with instability of coding microsatellites (or coding simple sequence repeats) due to deficient DNA mismatch repair. Microsatellite instability-high tumors like colorectal, endometrial, and gastric cancer generate peptides arising from these mutated and expressed microsatellites that possess high immunogenic properties [24,25,26]. Martin et al. identified hidden NOPs arising from structural variants in which regulatory elements from upstream protein-coding genes promote the expression and translation of previously unannotated genomic sequences located downstream of chromosomal rearrangement breakpoints. Moreover, these NOPs can generate immunogenic neoantigens that can bind to MHC-I molecules [27].

Interestingly, although most of the frameshift mutations are unique for each patient, Koster et al., after analyzing 10,186 TCGA tumor samples, have seen that neoantigens originated from frameshift mutations are shared by large groups of cancer patients [23].

#### 2.2.2. Aberrant Splicing and Non-AUG Initiation

Beyond genomic alterations, transcriptomic and translational dysregulation further diversify the repertoire of potential neoantigens. RNA editing events, alternative splicing, and aberrant transcriptional regulation collectively expand the cancer proteome, generating novel protein variants [28]. These processes not only diversify the cancer proteome but also generate immunogenic neoantigens through the production of aberrant peptide sequences [29,30].

Tumor-specific aberrant splicing events (neojunctions) generate a large and still underexplored class of potential neoantigens that can elicit immune responses and may be exploited for cancer immunotherapy. In a study by Kahles et al., the inclusion of neojunction-derived peptides as candidate antigens, in addition to single-nucleotide variant derived peptides, increased the proportion of samples with at least one mass spectrometry (MS)validated neoantigen from 30% to 75%. In addition, the splicing-derived putative neoepitopes have a high degree of recurrence, suggestive of their potential use in immunotherapeutic intervention [29].

An analysis of TCGA data revealed an average of 94 public neojunctions per tumor type, with consistent frequencies across samples and splice type variation [19]. Hierarchical clustering demonstrated that neojunction expression patterns are tumor-type specific, with a subset of neojunctions shared across multiple cancer types, suggesting pan-cancer therapeutic potential. In different cancers, including myeloid leukemia, cholangiocarcinoma, and gliomas, specific neojunction subsets correlated with isocitrate dehydrogenase (IDH) mutation status [19]. Mechanistically, neojunction formation can be driven by splicing factor mutations like SF3B1 [31], altered expression of RNA-binding proteins such as ACAP2, or by altered expression of wild-type splicing factors like SNRPD2 or SF3A3 [19]. These findings indicate that dysregulated expression of canonical splicing-related genes, and not only splicing factor mutations, can generate disease-specific neojunctions with potential as immunotherapy targets. Moreover, different studies have shown how neojunctions can generate neoantigens that are presented by the MHC. Kwok et al. identified T cell receptor (TCR) clones that specifically recognize and target neoantigens arising from aberrant splicing in GNAS and RPL22 [19]. Neoantigens with immunogenic potential were also identified by Merlotti et al., in human non-small cell lung cancer where they found tumor antigens derived from noncanonical mRNA splicing events between coding exons and transposable elements [32]. Together, these quantitative observations further support the idea that non-canonical transcriptional and translational events may generate recurrent antigenic peptides that could expand the repertoire of potential neoantigens.

Protein translation can also initiate at alternative start codons rather than the canonical AUG. Proteins and peptides have been identified from non-primary AUGs [33] as well as from near-cognate translation start sites that differ from AUG by a single nucleotide, being CUG the one with the highest efficiency after AUG [34]. In a proteogenomic study carried out in B-Cell Lymphoma cell lines by Ruiz Cuevas et al., they established that 40% of novel proteins started on an unannotated AUG and more than half of the MHC-associated peptides source proteins were translated from non-AUG near-cognate start codons [35]. These non-canonical translation mechanisms are also highly relevant for peptides derived from non-coding transcripts, including lncRNAs, which frequently rely on alternative initiation sites and unconventional translational regulation [35].

#### 2.2.3. Non-Coding RNAs as Antigenic Reservoirs

Less than 2% of the genome codifies proteins, whereas at least 75% is actively transcribed into ncRNAs [36]. The integration of high-throughput sequencing technologies has revealed that these transcripts can contribute to the production of previously unannotated peptides that expand the immunopeptidome. Among the different classes of ncRNAs, lncRNAs represent a particularly promising source of cryptic peptides with potential immunogenic properties, as discussed in the following section.

## 3. LncRNAs as a Source of Cryptic Peptides

Initially, lncRNAs were considered to lack protein-coding potential, and several studies revealed that these molecules were primarily associated with the regulation of both gene expression and some cellular homeostasis processes. However, subsequent analyses of their sequences showed that a significant fraction of them contain smORFs encoding peptides of less than 100–150 amino acids with different biological functions. These smORFs were frequently overlooked or misannotated, as they were considered not to conform to the minimal consensus ORF length of at least ~100 amino acids from the START to the STOP codon. Therefore, the main difficulty lies in identifying true functional peptides and excluding ‘transcription noise’ that is not translated [37,38].

As discussed in the previous section, non-coding transcripts can contribute to the generation of non-canonical antigenic peptides. Among them, lncRNAs represent a particularly relevant source due to their widespread expression in cancer and their potential to encode immunogenic peptides.

### 3.1. Biogenesis of lncPEPs: smORFs, Ribosome Association and Translation Mechanisms

It is known that lncRNAs can be transcribed by RNA polymerase II or other RNA polymerases and undergo post-transcriptional mechanisms in a similar way to pre--mRNA: the addition of 5′-CAP (5′-(7-methylguanosine (m^7^G)), polyadenylation at the 3′ end (3′ poly-A tail), splicing and folding RNA processes [39]. Following nuclear processing, these transcripts can either be retained in the nucleus or exported to the cytoplasm through the nuclear pore complex. Despite their similarity to mRNA, these lncRNAs tend to have in general fewer but larger exons, weaker internal splicing signals, and a greater distance between the 3′ splice site and the branch point compared to mRNAs [39,40].

Owing to these mRNA-like features, lncRNAs are capable of being recognized by ribosomes and therefore display the potential to be translated. However, assessing their protein-coding capacity is still challenging, because coding sequences are sometimes found inside introns or overlapping with exons of different genes. It should be noted that only about 39% of human lncRNAs interact with ribosomes, but this interaction does not necessarily imply that these lncRNAs can encode peptides [39,40,41]. Current evidence indicates that canonical smORFs present in lncRNA undergo translation processes through mechanisms similar to those described for mRNA. These lncRNAs share subcellular localization and translation efficiency levels similar to those observed for canonical mRNAs [9]. However, it is also known that there are smORFs in lncRNA that do not use the AUG codon as start codon. For example, GUG and CUG codons allow translation to start, being the CUG codon the most common start codon within lncRNA. In contrast, AGG, AAG, and AUA are rarely used [40,42].

In addition to the conventional mRNA-like biogenesis pathway attributed to lncRNAs, alternative lncRNA processing routes have also been described. As a result, lncRNAs lacking a canonical mRNA structure can also be translated. Several lncRNAs have been reported to lack a 5′-CAP, have a shorter or even absent poly-A tail, contain a triple helix structure at the 3′ end to prevent RNA degradation, or even have a small nucleolar RNA cap (snoRNA-capped). Moreover, the most abundant modifications present in lncRNA are the inclusion of modified nucleotides: N^6^-methyladenosine (m^6^A) and 5-methylcytidine (m^5^C); pseudouridine (Ψ) and inosine (I) [39]. These structural and chemical features have different effects on lncRNA expression, as those with lower polyadenylation and inefficient splicing are more susceptible to degradation by the RNA exosome complex, which partly explains their low expression. In addition, the presence of a short poly-A tail has also been linked to reduced translation efficiency [43]. However, studies on mRNA have revealed that pseudouridine incorporation has been linked to greater stability, and that in mammals, m^6^A modification could facilitate lncRNA translation [39].

An alternative mechanism allowing lncRNA translation is the presence of internal ribosomal entry sites (IRES). IRES elements are secondary or tertiary RNA structures that can recruit the 40S ribosomal subunit in the 5′UTR region. These elements can then facilitate the translation process through a 5′cap-independent mechanism, and RNA-binding proteins (RBPs) can regulate the function of IRES, allowing RBPs to bind to lncRNA to form functional ribonucleoprotein (RNP) complexes that promote translation [40,43,44].

In summary, all these events contribute to the generation of previously unannotated peptides that may subsequently enter antigen processing pathways, enabling their presentation by MHC molecules and recognition by the immune system.

### 3.2. Evidence of lncPEP Expression in Cancer

In the 49th version of the Encyclopedia of DNA Elements (ENCODE) Project Consortium (GENCODE), nearly 36,000 lncRNA genes and more than 191,000 lncRNA transcripts have been cataloged. Numerous studies have linked a wide variety of lncRNAs to different diseases, among which cancer predominates. This is because lncRNAs can play a role in the gene regulation of cellular processes, acting as oncogenes, tumor suppressor genes and recruiting chromatin remodeling agents, among other functions. Thus, lncRNAs and their correspondent lncPEPs have been linked to cancer development [36,41,43,45,46,47].

In colon cancer, lncRNA HOXB-AS3 is downregulated; It encodes a 53 aa peptide which is an antagonist of the splicing factor hnRNP A1. This peptide causes a decrease in pyruvate kinase synthesis and therefore a reduction in lactic acid production, which inhibits the proliferation of colon cancer cells. The decrease in these anti-cancer peptide is associated with cancer development [41]. In advanced colorectal cancer, the expression of lncRNA LOC90024 produces a 130 aa protein called small splice regulatory protein (SRSP) in advanced colorectal cancer, which can promote the proliferation, migration, and invasion of colon cancer cells. SRSP interacts with the splicing factor 3 (SRFS3) of mRNA and increases its binding to exon 3 of the Sp4 transcription factor transcript, inducing the formation of the long, cancerous Sp4 isoform and inhibits the formation of the short, non-cancerous Sp4 isoform. This is an example of alternative splicing as a regulatory mechanism promoted by a lncPEP [41,48]. The expression of 55 lncRNAS has been identified in SW480 and SW620 colorectal cancer cell lines. One of them, LINC00266-1, encodes for a 71 aa peptide, called RNA-binding regulatory peptide (RBRP), which regulates the binding of RNA-binding proteins (RBPs) to RNA. Among the RBPs is the N6-methyladenosine (m^6^A) reader IGF2BP1, which recognizes m^6^A in c-Myc mRNA, thereby increasing its stability. As c-Myc is an oncogene, RBRP promotes tumorigenesis and tumor metastasis [41,49].

In triple-negative breast cancer, two peptides derived from different lncRNAs are known to play a role in this disease. The first of the four smORFs present in LINC00665 is the only one that generates a micropeptide when translated. This 52-amino acid peptide, called CIP2A binding peptide (CIP2A-BP), binds directly to the CIP2A (cancerous inhibitor of protein phosphatase 2A) oncogene, preventing this oncogene from binding to the B56γ subunit of protein phosphatase 2A (PP2A), and thus maintaining the tumor-suppressing activity of PP2A and preventing tumor cell proliferation, tumor growth and metastasis [41,50]. In addition, the ASRPS peptide generated by the third smORF present in lncRNA LINC00908 appears to have an inhibitory effect. This 60 aa peptide acts as a regulator of the STAT3 peptide by decreasing its phosphorylation, which results in reduced expression of vascular endothelial growth factor (VEGF) and, therefore, in the blocking of blood vessel formation and angiogenesis. This lncRNA is downregulated in triple-negative breast cancer [41,51]. In ovarian cancer, it has been observed that the expression of the DDUP peptide, encoded in the CTBP1-DT lncRNA, is induced by DNA damage. This peptide contributes to the repair of DNA damage induced by cisplatin therapy. However, high expression of the DDUP peptide is associated with a worse prognosis of the disease, as patients who were resistant to this therapy had high expression of the lncRNA-derived peptide [52].

There are also peptides derived from lncRNA that do not exhibit exclusive specificity for a particular type of tumor tissue. This is the case of the MIAC micropeptide, encoded by lncRNA AC025154.2, which is expressed in renal, thyroid, prostate, lung adenocarcinoma, and colon cancer. However, the expression of this peptide appears to be dependent on the tumor context, MIAC showed significantly reduced expression compared to the levels of this peptide recorded in normal cells in adjacent tissues [53]. As some lncPEPs play different roles depending on the type of cancerous cell that is considered, it is crucial to describe the action of the each peptide within a particular cancer model [53,54]. The same occurs for the peptide Mitorregulin (Mtln), derived from the translation of LINC00116. This 56-amino acid peptide is part of the mitochondrial proteome and plays a role in cellular respiration [55]. In breast cancer cell lines treated with endoplasmic reticulum stress inducers, the absence of Mtln reduces cell viability; and in cervical cancer cell lines, cell proliferation and mobility appear to be dependent on its expression [52].

The existence of functional lncPEP regulating other cancerous cells is scarce. Therefore, further research is necessary to provide a deeper understanding of cancer biology, and the potential of lncPEPs as therapeutic targets [56].

### 3.3. Immunogenic Potential and Therapeutic Relevance of lncPEPs

As mentioned in the previous section, lncPEPs have been implicated in diverse biological functions and tumoral processes. Notably, a subset of lncRNA genes, upon translation into lncPEPs, can be subsequently processed into small antigenic peptides that are presented via MHC-I molecules. The presented antigens may triggers an immunogenic response mediated by CD8^+^ T lymphocytes, which can significantly delay tumor growth. Thus, the antigenic peptides derived from lncPEPs that are presented by MHC become part of the immunopeptidome and represent an emerging source of neoantigens [22,57,58].

LncRNAs, encoded lncPEPs and their derived neoantigens can exhibit varying degrees of tumor specificity. This is the case of the immunogenic peptides derived from MALAT1 and DANCR lncRNAs in different types of cancer [41,57,59,60]. This tumor specificity is particularly relevant in malignancies with low mutational burden, where mutation-derived neoantigens are limited and alternative antigen sources may provide new therapeutic opportunities. In this context, the immunogenic potential of neoantigens derived from lncPEPs opens a new avenue of research, as these peptides could be exploited for the development of cancer vaccines aimed at eliciting effective antitumor immune responses [57].

In the case of so-called ‘cold’ tumors, cryptic peptides derived from non-coding regions of the genome, including those originating from lncRNAs, may represent a potential additional source of targetable antigens. These tumors are characterized by a typically low rate of somatic mutations or low expression of classic neoantigens, an immunosuppressive tumor microenvironment (TME) due to lower expression of the immunosuppressive checkpoint, and a lower number of immune cells infiltrating the TME. These factors are often the reason why these ‘cold’ tumors are able to evade immune detection [15,61,62,63]. This immune escape could be reversed using cancer immunotherapies, including the use of non-canonical neoantigens for the design of neoantigen-targeted tumor vaccines. These therapies can improve T cell activation for antigen presentation by the immune system, converting a ‘cold’ tumor into a ‘hot’ one with an improved immune response, and increasing the efficacy of the treatments used against the disease [8,63,64,65,66].

In this context, lncPEPs are attractive targets due to their potential tumor specificity, broader genomic origin, and possible recurrence across patient subsets. Given that most of the human genome corresponds to non-coding regions, these sequences may constitute a large potential reservoir of previously unexplored antigenic peptides. However, the extent to which neoantigens derived from non-coding regions contribute to clinically relevant immune responses remains an active area of investigation. Some studies suggest that certain non-canonical peptides may recur across patient subsets, although further experimental validation will be required to determine the frequency and therapeutic relevance of such events. [8,61,67,68]. Together with their potential to elicit robust immune responses and the expansion of the non-canonical antigen landscape, these characteristics make cryptic peptides particularly attractive candidates for the development of cancer immunotherapies.

## 4. Antigen Processing and Presentation of lncPEPs

Antigens in their free or soluble form can be recognized by the immune system through antibodies or B lymphocytes. However, for these molecules to be recognized by T lymphocytes, they must be bound to a molecule of the MHC, known in humans as human leukocyte antigens (HLA). Within the adaptive immune system, there are two types of MHC responsible for recognizing and presenting the antigenic peptide on the cell surface. Each of them is activated depending on the origin of the antigen, such that MHC-I binds to intracellular or endogenous antigens, and MHC class II (MHC-II) recognizes and binds to extracellular or exogenous antigens. In the case of MHC-I, recognition of the peptide-MHC complex occurs via the cytotoxic CD8^+^ T lymphocyte receptor, while for MHC-II molecules, CD4^+^ T helper lymphocytes are responsible for this recognition. In addition to MHC-I and MHC-II, there is a third type of MHC, MHC-III, which does not participate directly in antigen presentation but contains information about proteins that may or may not participate directly in the immune response. As lncPEPs are of intracellular origin, this section focuses on the functioning of MHC-I [69,70].

The generation of lncPEPs alone is not sufficient to confer immunological relevance; these peptides must undergo antigen processing and presentation to become visible to the adaptive immune system. As discussed in the previous sections, non-canonical translation products derived from lncRNAs can expand the tumor immunopeptidome, but their immunogenic potential ultimately depends on their ability to be processed and presented by MHC molecules.

### 4.1. MHC-I Antigen Processing Pathway Revisited

**Figure 1:**
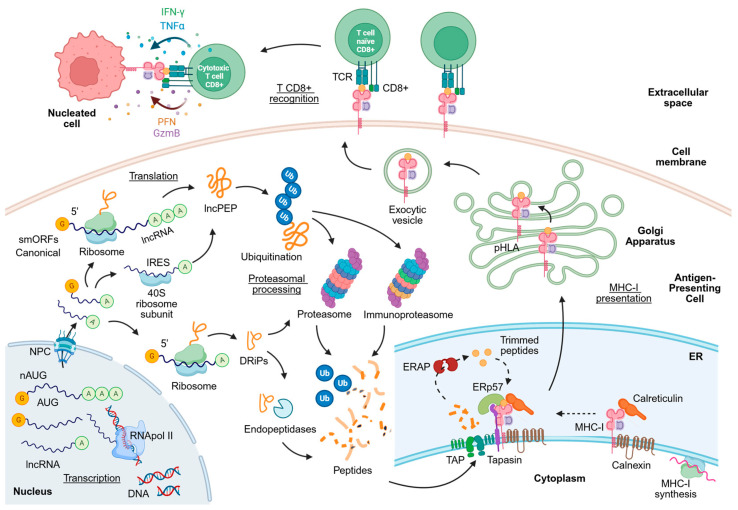
Major histocompatibility complex class I (MHC-I) Antigen presentation pathway. Transcription of DNA generates coding and noncoding transcripts, including long non-coding RNA (lncRNAs) with diverse structural features. These transcripts may undergo canonical translation or noncanonical initiation (e.g., non-AUG start codons, small open reading frame (smORFs), or internal ribosome entry site(IRES)-dependent mechanisms), producing lncRNA-derived peptides (lncPEPs). Stable lncPEPs can undergo ubiquitination and degradation by the proteasome or immunoproteasome, whereas unstable or defective ribosomal products (DRiPs) may also be processed by cytoplasmic proteases. The resulting peptides are transported into the endoplasmic reticulum (ER) via transporter associated with antigen processing (TAP) and further trimmed by endoplasmic reticulum aminopeptidase (ERAP). MHC-I molecules are assembled in the ER in association with the peptide-loading complex (including TAP, tapasin, ERp57, calnexin, and calreticulin), enabling stable peptide loading and formation of the peptide–HLA complex (pHLA) complex. The pHLA complex is transported through the Golgi apparatus to the cell surface via exocytosis, where it can be recognized by naive CD8^+^ T cells, leading to their activation and differentiation into cytotoxic T lymphocytes that mediate antigen-specific immune responses.

Endogenous proteins present in the cytosol are degraded primarily by the proteasome, a multicatalytic protease complex that generates peptide fragments of varying lengths. In inflammatory conditions, the constitutive proteasome can be replaced by the immunoproteasome, which exhibits altered catalytic activity and produces peptides with higher affinity for MHC-I molecules. The resulting peptides are subsequently transported into the endoplasmic reticulum (ER) through the transporter associated with antigen processing (TAP), a heterodimeric complex that selectively translocates peptides of appropriate length and sequence (Figure 1) [69].

Within the ER, peptides undergo further trimming by aminopeptidases such as ERAP1 and ERAP2 to achieve optimal size for MHC-I binding. Peptide loading occurs within the peptide-loading complex, which includes TAP, tapasin, calreticulin, ERp57, and MHC-I heavy chain–β2 microglobulin heterodimers. Once a stable peptide–MHC complex is formed, it is transported through the Golgi apparatus to the cell surface, where it can be recognized by TCRs expressed on CD8^+^ T lymphocytes. When a lymphocyte’s TCR binds to a specific MHC, i.e., when the TCR reacts to a specific antigen, it is activated, undergoes clonal expansion, and differentiates into a cytotoxic CD8^+^ T lymphocyte (CTL). This increases the number of T lymphocytes reactive to that antigen, which is detected as ‘foreign.’ These CTLs develop membrane-bound cytoplasmic granules containing proteins such as perforin and granzyme, which respectively form pores in the membrane and trigger apoptosis of the cell presenting the pHLA (peptide-HLA) complex on its surface, thereby triggering the cell-mediated adaptive immune response necessary for antigen elimination (Figure 1) [69,70].

Importantly, this antigen processing machinery does not discriminate between peptides derived from canonical proteins and those originating from non-canonical translation events [71]. Therefore, peptides encoded by lncRNAs, once synthesized in the cytoplasm, can enter the same processing pathway and be presented by MHC-I molecules, enabling immune recognition. Likewise, non-canonical translation can also generate unstable defective ribosomal products (DRiPs), short-lived proteins that can give rise to cryptic peptides through proteasome-independent mechanisms, such as cytoplasmic endopeptidases, after which they are loaded onto MHC-I molecules and transported to the cell surface for immune recognition (Figure 1) [72].

Although MHC-I molecules are constitutively expressed on the surface of almost all nucleated cells, not all peptides are able to trigger an immune response. This is due to the central tolerance property of self-antigens. This property is developed in the generating lymphoid organs (specifically in the thymus in the case of T lymphocytes), since foreign antigens that enter the body are usually captured by the peripheral lymphoid organs; and allows the elimination of those immature T lymphocytes or thymocytes that recognize self-antigens, thus preventing an immune response against the self. Since not all self-antigens are expressed in the thymus, there are a number of additional mechanisms, such as anergy, antigen presenting cell (APC) generation, or peripheral elimination of autoreactive lymphocytes, collectively referred to as peripheral tolerance, which allow for the control of those lymphocytes that find their reactive antigen outside the generating lymphatic organ, so as not to generate an autoimmune response [69,73,74].

In addition to the classical pathway of endogenous antigen presentation through MHC-I, it is also possible for cells that normally present antigens via MHC-II to present them through MHC-I molecules. This phenomenon, known as cross-presentation, occurs primarily in dendritic cells (DCs), a specialized type of APC capable of internalizing exogenous material, such as infected or tumor cells, through phagocytosis or endocytosis. Instead of being processed exclusively for MHC-II presentation, a fraction of these exogenous antigens can enter the MHC-I processing pathway, allowing peptides derived from external sources to be presented on MHC-I molecules. Through this mechanism, DCs can activate CD8^+^ cytotoxic T lymphocytes against antigens that are not synthesized within the presenting cell itself. In this way, DCs are able to simultaneously present antigens through both MHC-I and MHC-II, which are recognized by CD8^+^ and CD4^+^ T lymphocytes, respectively. [69,75,76].

### 4.2. Computational Prediction Challenges for Neoantigen–HLA Binding

Since the end of the 20th century, with the development of the Human Genome Project, biology has entered the era of omics thanks to the development of new high-performance technologies that enable the collection of large amounts of genomic, transcriptomic, and proteomic data. In recent years, advances in computational methods have made it possible to process and analyze all this vast amount of information more efficiently [77,78]. These technological advances have been particularly relevant for the identification of non-canonical peptides, including those derived from lncRNAs, within the expanding landscape of the tumor immunopeptidome.

Currently, the identification of peptides derived from lncRNA is possible thanks to the integration of information obtained from different experimental approaches such as Global Translation Initiation Sequencing (GTI-Seq), ribosome profiling, study and identification of canonical and non-canonical ORFs, and MS-based proteomics. The combined use of these methodologies can generate a large amount of data that needs to be processed using specific bioinformatic tools and advanced computational resources [41].

This increase in the amount of data obtained allows for an expansion of the search space, that requires the use of a large number of computational resources, which until recently were not so readily available. Furthermore, unlike classical coding regions, non-coding regions (including lncRNAs) are less annotated, and therefore there is less information about them in public databases. This is why it is often necessary to use customized or specific databases for each particular case, which allow the inclusion of sequences derived from lncRNA, ncORFs or other non-canonical variants, and which can be used to feed the bioinformatic tools employed [79,80]. Consequently, the prediction of peptide–HLA interactions for lncRNA-derived sequences presents additional challenges compared with canonical peptides, due to their reduced annotation, potential low expression levels, and unconventional translational features.

Remarkably, the algorithms used in the bioinformatic analyses and prediction tools are not yet perfectly optimized. The tools used for predicting peptide-HLA binding have been trained mainly with datasets enriched in a limited number of frequent alleles and canonical peptides, due in part to the limitations of current knowledge in the field of immunology. As a result, their generalization capacity may be reduced when, instead of using frequent data, less represented alleles or non-canonical peptide sequences are used. In addition, the predicted binding affinity does not always correlate with the formation of stable pHLA complexes or with the induction of an effective immune response. All these cases highlight the complexity of predicting the actual immunogenicity of a peptide [81,82].

These limitations in the models are largely due to the scarcity of experimental data, since the models cannot be efficiently trained and may therefore introduce biases in the predictions. For this reason, the use of these bioinformatics tools allows for an initial approximation, but experimental methodology is still necessary for the validation of the results obtained in silico. Iterative feedback between experimental data and computation algorithms will improve the performance of future predictor models, so they can be used for the development of personalized cancer immunotherapies [22,83]. Integrating computational prediction with experimental identification and functional validation strategies is essential to accurately characterize neoAgs-lncRNA with potential translational relevance.

## 5. Strategies for Identifying neoAgs-lncRNA

As discussed in the previous sections, the non-canonical origin of these peptides introduces additional complexity compared with mutation-derived neoantigens, making the development of robust identification strategies essential for their reliable characterization. The ability of lncRNAs to produce neoantigens can be studied thanks to advances in omics techniques, which allow ORFs to be identified and other non-canonical start codons to be considered. These techniques continue to be used today, as the current general strategy for identifying lncRNA-derived neoAgs is based on a combination of transcriptomic analysis, immunopeptidomics and bioinformatic predictions, integrated under a bioinformatic pipeline. This multi-omic approach allows the tumor immunopeptidome to be mapped, but it is still necessary to confirm the immunogenicity of neoAgs by performing functional validation tests so that, if positive, they can be used in personalized immunotherapies [64,84,85,86,87,88,89].

### 5.1. Transcriptomics and Translatomics (RNA-Seq, Ribo-Seq)

Within the integrated analysis for the identification of neoAgs, transcriptomics allows the creation of databases from data obtained through RNA-seq or Ribo-seq, in order to compare proteomic results with databases that include non-canonical regions, since most public databases only integrate canonical peptides encoded by classical exons [85,86,88].

RNA sequencing using next-generation sequencing (NGS) technology allows the entire transcriptome present in samples of interest to be analyzed, so that a specific and non-exclusive database of canonical transcripts can be created for use in peptide identification in proteomics studies. This technique also provides information on gene expression levels, allowing the selection of those more likely to generate cryptic peptides, as well as considering other genetic modifications [87,88,89].

Ribosome profiling is another methodology used in the discovery of neoantigens. This technique allows the identification of regions of the transcriptome that are active in translation, as it is based on the sequencing of mRNA fragments protected by ribosomes. This technique allows the recognition of codons translated in a specific reading pattern, as well as the identification of new ORFs, making it possible to identify translation outside canonical regions. In this way, by identifying and locating start codons, they can be integrated when generating a customized database [85,86,87,89]. This approach is particularly relevant for lncRNA-derived peptides, as many smORFs embedded within lncRNAs are not annotated in conventional databases and can only be detected through translatomic evidence.

**Figure 2:**
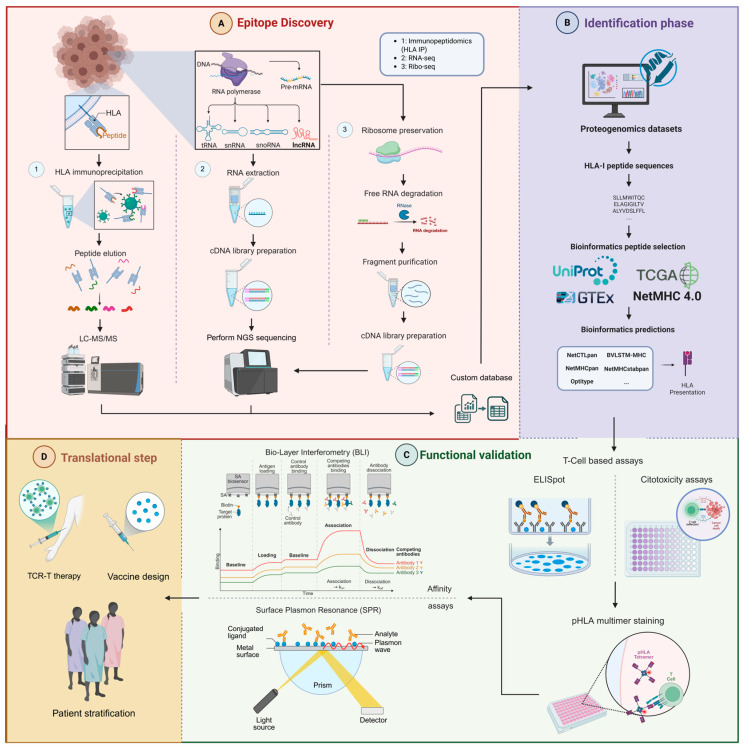
Integrated pipeline for the discovery and validation of HLA-restricted antigens. (**A**) In the epitope discovery phase, immunoprecipitation of HLA-peptide complexes (HLA immunoprecipitation), together with transcriptomic (RNA-seq) and translational (Ribo-seq) data, are integrated to generate a customized database of potential neoantigens. (**B**) In the identification phase, a proteogenomic approach is applied to identify HLA-associated peptide sequences, followed by prioritization using bioinformatics tools that predict HLA binding and immunogenic potential. (**C**) The functional validation phase includes T cell-based assays (such as cytotoxicity and ELISpot), pHLA multimeric staining, and TCR-pHLA affinity analysis (e.g., BLI or SPR) to confirm the presentation and immunogenicity of the selected peptides. (**D**) Finally, in the translational step, validated antigens can be used for patient stratification and the development of personalized therapeutic strategies, such as vaccines or adoptive TCR-T therapies.

Both RNA-seq and Ribo-seq are the two most commonly used methodologies for identifying non-canonical peptides in cancer using tumor samples (Figure 2A). However, whole genome sequencing (WGS) or whole exome sequencing (WES) are sometimes proposed as alternative approaches. The advantage of WES over WGS is that the former only covers 2–3% of the genome, making it cheaper than WGS, but it targets the coding genome, which limits its applicability in the identification of peptides derived from non-coding regions. However, the combination of RNA-seq and WES provides an optimal approach for HLA allele typing, which is necessary for studying the affinity between the peptide and HLA for the formation of the pHLA complex that can be recognized by a TCR [87,89].

Currently, there is a wide range of bioinformatic tools that allow a comprehensive exami-nation of the transcriptome in order to identify non-canonical ORFs, considering variant annotations, alternative splicing, or different start codons, and translation in all possible reading frames thus generating customized databases [41,86,90,91]. In addition, this research field is exponentially expanding.

### 5.2. Proteogenomics and Immunopeptidomics

Proteomic detection of neoantigens relies on MS and on customized non-canonical protein databases constructed from transcriptomic datasets for tumor specific antigens. This approach is known as proteogenomics, an integrative field that combines proteomic and genomic analyses [85,86,87]. In the context of neoAgs-lncRNA, these customized databases frequently include predicted smORFs and non-canonical ORFs derived from lncRNA transcripts (Figure 2B).

Fundamentally, there are two ways for MS data acquisition: data-dependent acquisition (DDA) and data-independent acquisition (DIA). DDA is widely used for the reliable identification of peptides, including non-canonical peptides and post-translational modifications. This mode is based on the fragmentation of a selected precursor according to different factors such as intensity and charge, generating high-quality MS/MS fingerprint spectra, but it has low reproducibility between samples. Therefore, for comparative or differential analyses, it is advisable to use the DIA method, which simultaneously fragments several precursors within the same *m*/*z* window and allows for quantification and sample reproducibility [87].

The use of bioinformatics software, neural networks, or machine learning tools can help to predict the binding capacity of a peptide identified by proteogenomics to a specific HLA, but immunopeptidomics should be used to confirm the data obtained through predictive models. Immunopeptidomics involves immunoprecipitation of pHLA complexes exposed on the cell surface for subsequent proteogenomic study by LC-MS/MS. This makes it possible to reduce spectral noise and thus achieve better sensitivity for the analysis of cryptic peptides, as these are generally present in smaller quantities than canonical peptides, and this, together with their small size, makes their identification more complex due to lower quality MS/MS spectra in shotgun-type proteomic analyses [35,87,89,92,93,94,95,96].

As explained before, the identification of peptides after MS acquisition often requires the use of customized databases. Using personalized databases constructed with three possible reading frames based on information from RNA-seq greatly expands the search space, thereby reducing the discriminatory power of the statistics used, such as the false discovery rate (FDR), by increasing the number of random matches and allowing the wrong assignment of a peptide detected by MS to a given sequence present in the used database. This leads to false positives and losing true peptide-spectrum matches because they fall outside the established FDR limit. To address this issue, it is common practice to introduce decoy sequences into databases. Decoys are artificial sequences, typically generated by reversing or shuffling the target protein sequences, that are not expected to be present in the biological sample. Because any match to a decoy represents a false identification by definition, the rate of decoy hits can be used to estimate and control the FDR, thereby improving confidence in peptide identifications [35,38,97,98].

Due to the small size of the neoantigens and the expansion of the search space, it is recommended not to use a global FDR in favor of a specific FDR for this subset, since the global FDR, calculated over the entire search space, may be too permissive for a particular subset and admit a higher number of false positives. This is because, unlike classical proteomics, where two or more unique peptides are usually required to validate a protein, for these neoantigens it is often possible just having one peptide-spectrum match, so it cannot reduce the false positive rate in this case. Some authors propose the use of two-stage FDR, based on performing an initial search between the MS/MS spectra and a reference database in order to eliminate those that match with a certain level of confidence, and then comparing the remaining MS/MS spectra to a second specific database that includes the variants or non-coding regions of interest that are absent from the first database, with the aim of improving the statistical estimation of the FDR for non-canonical sequences [98,99,100].

The use of data obtained through Ribo-seq also allows the size of the database used to be restricted, as it is only generated with RNA that is actually being translated and in the correct reading frame. Therefore, predictions made with this type of database improve statistical confidence levels, unlike those based on RNA-seq data [87,101,102].

However, although immunopeptidomics allows neoantigens of different origins to be identified, this does not imply that they are immunogenic. To determine this, functional assays such as ELISPOT for INF-g and cytokine secretion, flow cytometry for the study of T-cell activation and proliferation or pHLA multimers, and quantification of specific T cells, among others, would be necessary [87,101,102]. Therefore, functional validation remains essential to confirm the immunogenic potential of candidate neoAgs-lncRNA.

### 5.3. Bioinformatic Pipelines for Neoantigen Discovery

In addition to the bioinformatic tools available for transcriptomics and proteogenomics, there are also resources specifically focused on immunology and neoantigen discovery, some examples of which are given below (Figure 2B).

Firstly, there are tools as NetCTLpan [103] designed to predict peptide processing and cleavage in the proteasome, transport via the TAP protein and antigen presentation by the MHC-I. These tools are mainly trained to analyze peptides of 8 to 11 amino acids. Other bioinformatic tools developed, such as Optitype, arcasHLA, and seq2HLA, focus on HLA typing, as HLA is highly polymorphic and there appears to be a bias in terms of the alleles that are mostly bound to cryptic peptides. Thus, the probability of a peptide being presented by the HLA is affected by both the peptide sequence and the HLA allele. These tools, based on both RNA-seq and WES or WGS data, therefore help to identify the HLA alleles present in the samples analyzed so that it is subsequently possible to make predictions of binding affinity between the specific peptide and HLA allele in each case [104,105,106].

On the other hand, there are bioinformatic tools developed to predict the binding between candidate peptides and the MHC-I complex. Within this group, we can find artificial neural networks, such as NetMHC, a tool trained with chemical affinity data that predicts the affinity of the neoantigens under study for each of the MHC-I alleles; or NetMHCpan, which, in addition to chemical affinity data, includes information on peptide elution in mass spectrometry. BVLSTM-MHC is another tool in this group which, unlike others, eliminates the limitation of a specific peptide size [107,108]. Tools such as NetMHCstabpan allow the stability of the pHLA complex formed to be studied through the magnitude of the half-life. This stability seems to be particularly relevant when determining whether or not a peptide is immunogenic [107]. In addition, there are bioinformatic tools more focused towards the study of immune system cells, such as DeepTCR or NetTCR, developed to predict the binding specificity between a TCR and a given antigen [109,110].

There are also deep learning tools designed to predict the immunogenicity of cryptic peptides, such as DeepHLApan. In addition to predicting the probability of the neoantigen being presented by HLA in the tumor cell, DeepHLApan analyses the possible immunogenic potential of a pHLA complex to promote T cell activation. Within the Immune Epitope Database (IEDB), there are also various useful tools for predicting B and T cell epitopes [111,112].

Finally, these tools can be integrated into a pipeline covering transcriptomics, proteomics, and immunopeptidomics to perform a comprehensive analysis for the identification of new cryptic peptides. However, these resources often have certain limitations, because they have been trained with canonical peptides or the more common HLA alleles, so that predictions in the case of non-canonical peptides or alleles not encoded in the tool’s algorithm may not provide results close to those that would be obtained experimentally. Therefore, the need for more comprehensive datasets and the improvement of current prediction models represents a challenge for the development of personalized cancer immunotherapies [113,114,115,116].

## 6. Functional Validation of neoAgs-lncRNA Immunogenicity

As discussed in the previous sections, computational prediction and immunopeptidomic identification alone are insufficient to determine the biological relevance of neoAgs-lncRNA, making functional validation a critical step in the characterization pipeline. It is necessary to confirm their ability to generate an immune response, confirming not only immunogenicity but also the specificity of HLA-TCR recognition and the therapeutic potential of the candidates (Figure 2C).

### 6.1. T Cell–Based Assays for Neoantigen Validation

The immunogenicity of neoAgs-lncRNA depends on their ability to be presented on the cell surface as pHLA complexes and specifically activate CD8^+^ T lymphocytes. To evaluate this, functional assays based on T cells are used. These methodologies allow the analysis of cytokine secretion, proliferation, cytotoxicity, and clonal expansion, providing a comprehensive view of the immunotherapeutic potential of the proposed neoantigen.

T-cell activation assays are typically performed using in vitro T-cell priming analysis [117], cytokine production, proliferation and cytotoxicity assays [102,118,119]. These approaches are essential for demonstrating that neoAg-lncRNA is not only immunogenic but also capable of recruiting a specific TCR repertoire. In vitro priming of T cells consists of activating naive T lymphocytes to make them functional effectors. In this way, for example, lymphocytes are primed with different molecules and then co-cultured with antigen-presenting cells (usually dendritic cells, although it can also be monocytes and B cells) [118], which can be modified to present an antigen of interest. This allows T cells to be functionally characterized through the expression of cytotoxins or cytokine secretion [117].

Neoantigen-induced activation can be quantified using cytokine production assays, typically interferon-gamma (IFN-γ), such as ELISpot or ELISA [120,121]. ELISA assays allow both the measurement of cytokine levels in media supernatants generated under T cell stimulation, and the identification of which cytokines are activated, providing information on the quality of the response. On the other hand, ELISpot provides information about the number of cytokine-producing cells, which is a more informative parameter when measuring the immunogenicity of an antigen [102,118,119]. Cytokines can also be stained intracellularly and measured by flow cytometry. Two methods are commonly used for this purpose: incorporating radioactive nucleotides into the DNA of dividing cells, which allows for tracking; and methods based on the dilution of a fluorescent dye, for example carboxyfluorescein succinimidyl ester, so that the fluorescence decreases by half with each round of cell division, which can be monitored by flow cytometry. This second approach is the most commonly used nowadays. Moreover, these techniques also allow staining of other surface markers, which makes it possible to differentiate cell types [102].

Other option is to use pHLA tetramers with the peptide of interest, labeled with a fluorescent dye [102]. pHLA tetramers are multimeric complexes composed of four peptide–MHC molecules bound together—commonly through a streptavidin–biotin scaffold—and conjugated to a fluorochrome, which increases the overall binding avidity to T-cell receptors (TCRs). This multimerization overcomes the intrinsically low affinity of monomeric peptide–MHC interactions with TCRs, enabling stable binding and the direct detection of antigen-specific T cells by flow cytometry or microscopy. Therefore, adding them to a T lymphocyte culture and subsequently performing cytometry allows the detection of those peptides that function as immunogenic ligands [122]. This approach has inconvenients, such as the limited availability of fluorochromes for detecting multimers and the difficulty of generating pHLA monomers. However, progress has been made in the development of technologies that overcome these challenges, including the development of HLA ligands that are released when exposed to UV light, allowing another epitope of interest to be used, or the use of fluorochrome combinations that allow for the evaluation of a greater number of T cell specificities [122]. In this way, by directly exposing CD8^+^ T cells to their antigens, it is possible to see whether they are activated through the secretion of molecules that report this process, such as IFN-γ, or directly through cell proliferation, when clonal division occurs, or through labeling, using pHLA tetramers, and fluorescence-activated cell sorting (FACS), even allowing activated T cell counts to be made, enabling the immunogenicity of pHLA to be evaluated [120,121,123].

### 6.2. TCR–pHLA Interaction Studies (SPR, BLI, Multimer Staining)

The molecular interaction between the TCR and the pHLA complex is essential for characterizing antigen recognition by T cells. Various methodologies are used for this purpose, such as staining with pHLA multimers, which allows the detection and quantification of T cells specific for certain antigens, as explained in the previous subsection.

However, in order to analyze TCR-pHLA interaction and its specificity, it is necessary to know parameters such as association and dissociation constants, affinity, and stability, in addition to verifying pHLA recognition in tumor cells and APCs. This requires other types of assays, including surface plasmon resonance (SPR) or biosensor interferometry (BLI) [124,125,126]. These techniques allow high sensitivity, the possibility of direct, quantitative measurement in real time, and the ability to test several antigens in parallel. In addition, they are valid for low or moderate affinity values, equilibrium dissociation constant (Kd) values in the micromolar range, or tags that do not modify the conformation of the biomolecule or pose a steric hindrance to be considered [73,124].

In SPR, a shining light is projected onto a glass prism covered by a thin layer of metal (gold or silver) and the variation in the refractive index near the metal surface is measured when one molecule binds to another. Part of the incident light is not reflected and is lost as evanescent waves, which cause oscillations (resonance) of the electrons (plasmon) in the metal. When the interaction occurs, in this case between TCR and pHLA (one being in a stationary phase on the metal and the other in a mobile phase), the mass on the metal surface increases, changing the angle at which the surface plasmon resonance occurs to change, resulting in a real-time signal [127]. BLI is based on measuring changes in optical interference when two molecules bind at the tip of an optical fiber, immobilizing the ligand at the tip of the biosensor (the optical fiber). When both molecules bind, the thickness of the biological layer increases, altering the interference pattern of the light reflected in the biosensor. Both techniques allow affinity and binding kinetics to be measured in real time [124,125,128].

### 6.3. Specificity, Affinity and Functional Relevance

To validate the immunogenicity of a cryptic peptide, it is necessary to go beyond the detection of immune activation, as this alone is not sufficient to predict the biological relevance of TCR-pHLA recognition [129,130]. It is necessary to integrate quality criteria such as molecular specificity, functional affinity, and functional relevance to see the real impact of the interaction.

Specificity refers to the ability of a given TCR to differentiate between antigens presented by the same HLA molecule. Although TCRs are capable of detecting and binding to several antigens, effective recognition depends on interactions in the complementarity-determining regions (CDRs) and residues of the pHLA complex [131]. It can be evaluated experimentally using comparative binding assays between different antigens, SPR, BLI, or structural analysis (such as crystallography) to identify critical molecular contact points. This is important because variations in certain peptide residues can significantly change the rates of association and dissociation, as well as the stability of the complex [132].

In terms of affinity, Kd, the dissociation rate, and the average lifetime of the complex formed are particularly relevant, as they are related to the potency of immune signaling [130], although the cellular context in which it occurs must be taken into account [133].

Functional validation is essential to understand the biological relevance of TCR-pHLA interaction, since the correlation between affinity and function is not always linear [134]. Excessively high affinities may alter antigen discrimination and increase the risk of cross-reactivity, while intermediate affinities may be optimal for maintaining specificity and effector efficacy [129,130]. To this end, cell assays are performed to evaluate cytokine production and the expression of cell activation or proliferation markers [121,129].

Therefore, the complete characterization of antigen recognition requires an integrated approach that includes quantitative biophysical analysis to determine kinetic parameters and affinity; evaluation of molecular specificity, including cross-reactivity studies; and cellular functional validation, demonstrating that recognition of neoantigen presented by APCs is capable of inducing an adequate effector response. Importantly, given their non-canonical origin, lncRNA-derived peptides require confirmation that they are naturally generated and presented in tumor cells under physiological conditions. This typically involves integrating evidence of translation with direct detection of pHLA complexes, thereby establishing the endogenous nature of the candidate neoantigen. In addition, comparison between tumor and normal tissues is essential to demonstrate tumor specificity and minimize the risk of off-target toxicity, particularly for therapeutic applications involving engineered T cells.

Together, this multidimensional approach allows us to determine whether TCR and pHLA binding occurs and the specificity of that interaction, assessing whether it is sufficiently stable and functionally relevant for the validation of neoantigens as potential immunotherapeutic targets.

## 7. Advanced Preclinical Models to Evaluate neoAgs-lncRNA-Targeted Immunotherapy

Following the functional validation strategies described in the previous section, advanced preclinical models are required to assess the therapeutic potential of neoAgs-lncRNA in biologically relevant contexts. The context in which tumor initiation, progression, and metastasis occur is important. TME is a specialized complex ecosystem in continuous change, comprising a set of different cells that are relevant for tumor progression and migration, as well as for resistance to chemotherapy [135]. For these reason the potential of neoantigens derived from lncRNAs (neoAgs-lncRNAs) must be evaluated in models as close as possible to clinical reality [136].

### 7.1. Limitations of 2D Cultures in Immunotherapy Research

In the case of cancer immunology, it is particularly difficult to obtain a suitable model due to the excessive complexity of the immune system, which presents a multitude of cell types, including several subtypes, as well as a wide spectrum of activation states [136]. 2D cultures have several advantages including low cost, high throughput capacity, and the possibility of using human cells to study human diseases [136]. However, the conditions in which the cells grow are still very different from those in the physiological TME, a complex and heterogeneous mixture of cell types and extracellular matrix, and a growing number of studies show that 2D culture systems alter both phenotype and cell physiology [136].

It has been demonstrated that 2D systems cause cells to perform bioactivities that differ from those they perform in vivo. The main limitations encountered when studying the TME include changes in: cell morphology, since the shape of each cell is altered, as well as loss of polarization; the genetic profile with adhesion, cell proliferation, and expression of survival genes is modified compared to in vivo conditions; basic tumor heterogeneity, which does not allow for clear observation of proliferation gradients, drug penetration, or differences in cell mobility [135].

One of the main characteristics of tumor cells is their rapid growth rate and loss of contact inhibition, which is a mechanism that causes mammalian cells to grow in a monolayer. This means that once the entire surface is occupied, tumor cells begin to stack up, which is a problem because it causes cell death. Similarly, in the case of co-culture with TME cells, being subject to contact inhibition affects the ratio of cells of each type [135]. Various studies have shown how proliferation is affected: 2D breast cancer cultures stopped growth after 7 days, while 3D cultures continued to grow for up to 13 days. In addition, cell morphology was affected, with 3D cultures more closely resembling that found in vivo [137,138].

Also cell migration and mobility are affected in 2D cultures, since in these cultures the main mechanism of migration is based on polarized signaling and mechanical aspects such as adhesion forces and cytoskeletal tension, whereas in tumor cells use more complex mechanisms involving the extracellular matrix [135,139]. Furthermore, expression of membrane receptors is higher in 3D models [140].

### 7.2. 3D Tumor Models: Spheroids, Organoids and Co-Culture Systems

3D models are an innovative and very useful tool, as they allow the TME to be accurately and realistically stimulated compared to monolayer (2D) cultures. They have structures that are more similar to the in vivo environment and are susceptible to changes in oxygen gradients, growth factors, and cell–cell and cell-extracellular matrix communication, compared to monolayer cultures [141,142]. When discussing 3D cultures, it should be noted that there are several alternatives with advantages and disadvantages among them. Three of the most commonly used 3D cultures are spheroids, organoids, and 3D co-cultures [141,143].

Spheroids consist of 3D cell aggregates that grow floating in the culture medium, and which are generated from established cell lines or patient-derived cells [144]. They try to replicate the architecture of a tumor in vivo, so they are often used in studies of drug resistance and tumor invasion [141]. The advantage of spheroids over other 3D cultures is that they are easily accessible. Since they simulate characteristics of the TME such as hypoxia, latency, and drug resistance, they are used for drug screening [136]. However, cellular homogeneity limits their use in immunotherapy studies, although they are useful for evaluating the penetrance of different therapies and the infiltration of immune cells into the tumor [136].

Organoids are 3D structures derived from adult stem cells, induced pluripotent stem cells, or cancer stem cells, which are cultured in extracellular matrix-based media, developing various cell types and recreating the corresponding tissue at many levels. This type of 3D culture is more close to the architecture of the organ under study, highlighting its heterogeneity and physiological complexity [144]. There are two distinct types of organoids: those that depend on scaffolding systems, such as those based on polymer hydrogels (synthetic or natural) or on the cellular matrix, and those that are independent of them [145]. They are more expensive and generally more difficult to establish and propagate than spheroids [141]. Organoids are particularly interesting for studying antigen presentation, as tumor-derived organoids have been shown to produce tumor antigens that can be captured and processed by dendritic cells, which subsequently present them to and activate CD8^+^ T cells [136]. This feature is especially relevant for evaluating neoAgs-lncRNA, as it enables the study of endogenous antigen processing and presentation in a context that closely resembles tumor physiology.

Finally, 3D co-culture models consist of organoids or spheroids cultivated alongside other types of cells, such as those of the immune system. This strategy allows different interactions to be evaluated and immunotherapies to be tested [141]. For example, the joint cultivation of dendritic cells, CD8^+^ cells, and tumor cells allows to measure cytotoxicity and evaluate the efficacy of T cells expressing chimeric antigen receptors against tumor cells. Furthermore, when testing therapies such as CAR-T, it allows the identification of new antigens and the evaluation of tumor infiltration under conditions very similar to those found in vivo [136].

### 7.3. Assessing T Cell Infiltration and Cytotoxicity in 3D Models

Cytotoxic CD8^+^ T cells have very potent antitumor activity, killing cancer cells and organizing a major immune response by releasing cytokines. Therefore, the infiltration capacity of this type of cells is a very important marker in patients [146]. One of the advantages of 3D models over 2D models is that they allow for the evaluation of T cell infiltration in the tumor, which is a vital aspect when making prognoses for patients. The infiltration of these cells is associated with a better prognosis, and it also allows for the evaluation of different therapies that may have a better response when applied [146,147]. The easiest way to achieve this approximation is by co-culturing the organoids with immune cells, either by adding them directly to the medium or by culturing them together [145]. Analysis and visualization techniques that account for the complete three-dimensional structure and spatial aspects of the culture should be employed to evaluate T cell infiltration in models such as tumor organoids [146,148].

Although T cell infiltration can be assessed using three-dimensional imaging techniques, it is also possible to indirectly estimate the degree of spheroid invasion by counting the number of “spines” or invasive cell projections. These structures reflect active invasive behavior of tumor cells, associated with local degradation of the extracellular matrix and the generation of new tumor-matrix interfaces. This process increases the effective surface area of the spheroid and modifies the local microenvironment, favoring the exposure of chemotactic signals and facilitating the migration and penetration of T cells into the 3D model [143,148,149].

Some of the techniques used for this purpose include imaging by immunofluorescence staining, immunohistochemistry, and confocal imaging, which allow visualization of cell invasion and cell–matrix interactions. For example, in some of the protocols described, proteins or molecules of interest can be detected while preserving the spatial position of the spheroids by Lin et al. [143]. In the method described, the spheroids are embedded in a hydroxyethyl agarose gel inside an agarose mold, so that the gel retains the spheroids at the bottom of the microwells. Next, a horizontal sectioning is performed in series with the bottom of the mold. This approach differs from conventional immunohistochemistry sectioning of spheroids, which involves collecting cells before their inclusion in hydroxyethyl agarose processing gel, which poses a risk due to the possibility of altering the spheroids, thus losing their spatial arrangement. This approach preserves the three-dimensional architecture of the spheroid and allows the spatial distribution of infiltrated T cells to be analyzed using imaging techniques. Complementarily, flow cytometry can be used to characterize CD8^+^ T cell populations and evaluate cytotoxicity parameters, although this technique requires the disruption of the 3D model and therefore does not preserve spatial information [143].

### 7.4. Engineering T Cells Targeting lncPEPs (TCR-T, CAR-T Perspectives)

As explained, by using 3D models, it is possible to generate a microenvironment that more accurately reproduces the characteristics of the tumor in vivo, which is particularly relevant in the preclinical evaluation of cellular immunotherapy strategies. In this context, 3D models are an optimal platform for studying the efficacy of specific tumor-infiltrating lymphocytes (TILs) against neoAgs-lncRNAs, taking into account tumor infiltration, cytotoxicity, and the spatial heterogeneity of the immune response.

Adoptive immunotherapy strategies based on genetically modified T cells mainly include CAR-T and TCR-T therapies [150,151]. CAR-T cells express a chimeric receptor capable of recognizing surface antigens independently of HLA, making them particularly effective against highly expressed membrane antigens. However, this approach has limitations for the recognition of neoAgs-lncRNAs, since these usually correspond to intracellular proteins processed and presented in the context of the major histocompatibility complex [152].

TCR-T involves genetically modifying T lymphocytes to express a specific TCR capable of recognizing a tumor peptide presented by HLA molecules on the surface of cancer cells [153]. Another alternative could be to generate ex vivo growth of TILs from a tumor [150]. Three main methods are required for gene edition: viral transduction (with lentiviruses being the most common method), mRNA electroporation (which has transient expression), and the most novel method, knock-in using CRISPR [150,154]. By producing modified TILs expressing a specific TCR against neoAgs-lncRNA, 3D models are used to test their infiltration capacity and cytotoxicity in the tumor, as well as to assess the spatial effects on their activity [143,148]. To evaluate infiltration, tumor spheroids are co-cultured with T lymphocytes and evaluated using confocal microscopy with fluorescent markers or time-lapse imaging. Cytotoxicity can be assessed by measuring the proportion of dead cells following co-culture using live/dead cell dyes, the release of lactate dehydrogenase (LDH) into the medium as an indicator of cell damage, or analyzing caspase activity as a marker of T cell-induced apoptosis [143,155].

In conclusion, the integration of 3D models derived from patients and adoptive immunotherapy treatments opens the door to the selection of more effective designed TCRs and to prediction of personalized patient response. These advances provide a foundation for translating neoAgs-lncRNA strategies into clinical applications, as discussed in the following section.

## 8. Selection and Functional Characterization of Immunogenic lncRNAs

The identification of lncRNA-derived peptides with immunogenic potential requires not only computational prediction and proteogenomic detection, but also a mechanistic understanding of the biological context in which these molecules are generated and recognized by the immune system. While previous sections have focused on describing identification pipelines and the functional validation of neoAgs-lncRNA candidates, this section addresses strategies for prioritizing and functionally characterizing immunogenic lncRNAs within their biological and immunological contexts. An additional critical layer involves determining which lncRNAs are most likely to produce clinically relevant immunogenic peptides, which requires integrating spatial biology, functional genomics, and immunological readouts to connect lncRNA expression, peptide production, and immune recognition within the tumor ecosystem. In this context, emerging technologies such as single-cell sequencing, spatial transcriptomics, and CRISPR-based perturbation platforms provide new opportunities to dissect the functional relevance of lncRNAs in cancer and their contribution to the immunopeptidome. These approaches grant the identification of lncRNAs that are not only translated into peptides but also associated with immune activation, tumor vulnerability, or therapeutic response.

### 8.1. Spatial and Single-Cell Approaches to lncRNA Prioritization

As previously mentioned, tumors exhibit profound cellular and spatial heterogeneity, which strongly influences antigen presentation and immune recognition. Bulk transcriptomic analyses can identify differentially expressed lncRNAs, but they fail to capture the cellular specificity and microenvironmental context that determine whether a given lncRNA is immunologically relevant. Single-cell RNA sequencing (scRNA-seq) has therefore been recognized as a powerful tool for resolving lncRNA expression at the cellular level, supporting the identification of tumor- or cell-state-specific transcripts that may encode immunogenic peptides. Integration of single-cell, spatial, and immunopeptidomic datasets provides a multidimensional framework for prioritizing lncRNAs that are both tumor-specific and immunologically active, reducing false positives derived from purely computational prediction pipelines.

Single-cell approaches enable integration of lncRNA expression with immune phenotypes, including T-cell infiltration, TCR repertoire diversity, activation states, and exhaustion signatures. Integrative immunogenomic analyses have demonstrated that specific lncRNAs expression correlates with the abundance and functional states of tumor-infiltrating immune cells across multiple cancer types, supporting their role as modulators of the tumor immune microenvironment rather than passive transcriptional byproducts. For instance, pan-cancer studies have identified lncRNA modifications associated with immune cell infiltration patterns and specific molecular subtypes with distinct immunological characteristics, highlighting their potential as regulators of immune–tumor interactions [156]. Furthermore, computational platforms integrating transcriptomic and immune-cell–resolved data have revealed lncRNA signatures that are predictive of immune infiltration and clinical outcomes, including associations with cytotoxic lymphocyte activity and response to immunotherapy [157]. Consequently, lncRNAs enriched in tumor cell populations that coexist with activated cytotoxic lymphocytes may represent candidates with higher immunogenic potential. Conversely, lncRNAs associated with immune-evasive phenotypes could identify mechanisms of resistance and novel therapeutic targets.

Spatial transcriptomics further expands this analysis by preserving tissue architecture and enabling the mapping of lncRNA expression within tumor niches. This is particularly relevant for neoantigen discovery, since antigen presentation and immune recognition are influenced by spatial proximity between tumor cells and immune infiltrates. Spatially resolved multi-omic analyses have demonstrated that immune activation occurs within defined intratumoral niches, such as tertiary lymphoid structures (TLS), where coordinated interactions between tumor cells, stromal populations, and immune subsets shape antitumor responses. For example, integrated single-cell and spatial transcriptomic profiling has revealed specialized TLS microenvironments containing stem-like CD8^+^ T cells, B cells, and supportive stromal populations that promote tumor cell apoptosis and enhance immunotherapy responsiveness, highlighting the functional importance of spatial immune–tumor interactions [158]. Similarly, spatial transcriptomic studies across multiple cancers have shown that TLS organization and immune-cell positioning at tumor borders correlate with antigen presentation, clonal expansion, and improved clinical outcomes, supporting their role as local sites of immune priming and effector function [159]. Therefore, spatial approaches can identify lncRNAs expressed within regions with active immune engagement, such as tertiary lymphoid structures or immune-inflamed tumor borders, thereby prioritizing candidates with functional immunological relevance.

### 8.2. Functional Interrogation Using CRISPR-Based Strategies

While expression data provide valuable clues, demonstrating functional relevance requires perturbation experiments. CRISPR-based technologies have revolutionized the study of non-coding RNAs by enabling targeted disruption, activation, or repression of lncRNA loci. These tools allow researchers to directly evaluate the impact of specific lncRNAs on tumor biology, peptide production, and immune recognition.

CRISPR-Cas9 knockout approaches can disrupt smORFs embedded within lncRNAs or delete entire lncRNA loci, enabling assessment of their contribution to antigen presentation. Loss-of-function studies combined with immunopeptidomics can determine whether eliminating a lncRNA candidate reduces the abundance of specific antigens presented by HLA molecules, providing direct evidence of peptide origin. Indeed, CRISPR-based functional screening has uncovered the translation and biological relevance of cryptic ORFs encoded within lncRNAs in cancer cells, demonstrating that disruption of these loci can alter cellular phenotypes and protein outputs derived from non-canonical regions [160]. Similarly, CRISPR interference (CRISPRi) and CRISPR activation (CRISPRa) systems allow modulation of lncRNA transcription without altering genomic structure, which is particularly useful for studying regulatory lncRNAs with overlapping genomic regions. Genome-scale CRISPRi screens targeting thousands of lncRNA loci have revealed widespread functional roles for non-coding transcripts in cell growth and context-specific phenotypes, highlighting the utility of transcriptional perturbation approaches for dissecting lncRNA function [161].

High-throughput CRISPR screening platforms further enable the systematic identification of lncRNAs that influence immune sensitivity. For instance, pooled CRISPR screens in tumor cells co-cultured with cytotoxic T lymphocytes can reveal lncRNAs whose disruption enhances or reduces immune-mediated killing. Such functional screens can identify lncRNAs involved in antigen processing pathways, interferon signaling, or immune evasion mechanisms, thereby linking lncRNA biology to immunogenic outcomes. Indeed, genome-wide CRISPR loss-of-function screens performed under immune selective pressure have identified tumor-intrinsic regulators of sensitivity to T-cell-mediated cytotoxicity, including genes controlling interferon-γ signaling and antigen presentation pathways that modulate immunotherapy response [162]. More recently, multimodal pooled CRISPR perturbation approaches combined with single-cell transcriptomic profiling have enabled the dissection of tumor immune-evasion programs at high resolution, revealing coordinated regulatory networks that determine susceptibility to immune attack and resistance to checkpoint blockade [163].

Importantly, CRISPR-based approaches can also distinguish whether the functional effect of a lncRNA is mediated by its RNA transcript or by its encoded peptide. This distinction is critical for understanding mechanistic pathways and for designing therapeutic strategies targeting either transcriptional regulation or peptide presentation. Indeed, functional CRISPR-Cas9 screening has revealed that some lncRNA loci exert biological effects through previously unrecognized encoded lncPEPs or other microproteins rather than through RNA-mediated regulatory mechanisms, pointing to the importance of experimentally dissecting coding versus non-coding functions [164]. Similarly, mechanistic studies of smORF-containing lncRNAs have shown that targeted disruption of peptide-coding regions can phenocopy deletion of the entire transcript. In contrast, mutation of RNA structural elements may produce distinct outcomes, supporting the concept that lncRNA loci can harbor dual functional modalities involving both RNA and peptide activities [165,166]. Together, these approaches enable precise mechanistic dissection of lncRNA biology and help determine whether therapeutic targeting should focus on transcript regulation or antigenic peptide presentation.

### 8.3. Linking lncRNA Biology, Peptide Production and Immune Recognition

A main challenge in the field is establishing a causal link between lncRNA expression, peptide generation, and immune recognition. The presence of a smORF and ribosome association does not necessarily imply that a peptide is processed and presented in sufficient quantities to trigger T cell responses. Therefore, integrated experimental frameworks are required to connect these biological layers. Systems biology approaches that combine multi-omic datasets with immune profiling may help elucidate these relationships and identify lncRNAs positioned at key regulatory nodes controlling immune pathways, thereby prioritizing candidates with higher translational potential. Such integrative strategies are essential for defining the biological continuum from lncRNA expression to functional immune activation and for distinguishing biologically relevant neoantigens from translational noise [167,168].

Another important aspect is the regulatory role of lncRNAs in immune regulatory pathways independent of peptide production. Some lncRNAs modulate antigen presentation machinery, interferon responses, or immune checkpoint expression, indirectly altering tumor immunogenicity. In such cases, both the regulatory RNA function and the encoded peptide may contribute to immune responses, highlighting the dual biological nature of certain lncRNAs. For example, the lncRNA LINK-A has been shown to suppress tumor antigen presentation by promoting degradation of components of the peptide-loading complex, thereby reducing MHC-I surface expression and impairing antitumor immunity [169]. Similarly, the interferon-stimulated lncRNA INCR1 regulates IFN-γ signaling and PD-L1 expression in tumor cells, and its silencing enhances sensitivity to cytotoxic T-cell–mediated killing, demonstrating how lncRNAs can control immune evasion pathways independently of peptide production [170].

### 8.4. Biomarker Potential of Immunogenic lncRNAs

Beyond their role as neoantigen sources, immunogenic lncRNAs may serve as biomarkers for patient stratification, prognosis, and prediction of therapeutic response. Because many lncRNAs exhibit tissue- and tumor-specific expression patterns, they are attractive candidates for non-invasive diagnostics and precision oncology.

Expression levels of certain lncRNAs have already been associated with immune infiltration, tumor progression, and survival outcomes across multiple cancer types. When linked to peptide presentation, these molecules could function as biomarkers predicting responsiveness to immunotherapies such as immune checkpoint blockade, personalized vaccines, or adoptive T-cell therapies. For example, tumors expressing lncRNAs that generate highly immunogenic peptides may be more susceptible to TCR-based therapies. Indeed, integrative analyses have identified lncRNA signatures associated with tumor-infiltrating lymphocyte abundance and clinical outcomes, demonstrating that lncRNA expression patterns can stratify patients according to immune activity and prognosis [157]. Furthermore, clinical studies have shown that specific lncRNAs, such as NEAT1, correlate with patient responses to immune checkpoint inhibitors, supporting their potential utility as predictive biomarkers of immunotherapy efficacy [157]. Furthermore, clinical studies have shown that specific lncRNAs, such as NEAT1, correlate with patient responses to immune checkpoint inhibitors, supporting their potential utility as predictive biomarkers of immunotherapy efficacy [171]. Additional examples reinforce this concept, including lncRNA signatures associated with interferon signaling and immune checkpoint expression that predict survival and immune infiltration across tumor types [172], as well as immune-related lncRNA risk models linked to CD8^+^ T-cell activity and therapeutic responsiveness in cancers such as melanoma and lung cancer [173].

Liquid biopsy approaches, including circulating tumor RNA analysis, may also enable monitoring of lncRNA expression dynamics during treatment. This could provide real-time information on tumor evolution, immune escape mechanisms, or treatment efficacy. Circulating lncRNAs are particularly attractive biomarkers because they can be detected in blood or other biofluids, reflect tumor biological processes, and allow repeated sampling over time. Indeed, studies have demonstrated that circulating ncRNA profiles correlate with tumor progression, therapeutic response, and drug resistance across multiple cancer types, supporting their potential for longitudinal disease monitoring [174]. Furthermore, extracellular vesicle–associated lncRNAs have emerged as stable and informative biomarkers that capture tumor-immune interactions and treatment-related changes, with evidence suggesting their utility for patient stratification and prognosis assessment in precision oncology settings [175].

However, several challenges remain before clinical implementation. Tumor heterogeneity, variable expression across patients, and limited annotation of lncRNAs complicate biomarker validation. Standardization of detection methods, reproducibility across cohorts, and integration with clinical parameters are necessary to translate these outcomes into clinical practice. Overall, the combination of spatial transcriptomics, functional genomics, and immunological assays provides a complete framework for identifying and characterizing immunogenic lncRNAs. These strategies bridge the gap between molecular discovery and translational application, facilitating the development of lncRNA-based immunotherapies and precision medicine approaches driven by biomarkers.

## 9. Discussion

Cancer development encompasses a broad range of molecular mechanisms, including transcriptional dysregulation of non-coding transcripts as lncRNAs. As a result, lncRNAs have emerged as promising source of non-canonical tumor neoantigens. In the following section the relevance and challenges of lncPEPs as a source of neoantigens will be discussed.

### 9.1. The Need for Highly Immunogenic Antigens and TILs in Cancer

TME plays a decisive role in tumor development. In the early stages of a tumor, immune cells and stromal components are recruited and activated, creating an anti-tumor inflammatory environment [176]. However, in more advanced stages of the tumor, an immunosuppressive TME is generated, which favors the development of cancer. In this pathological context, where the environment hinders effective antigen presentation and cytotoxic T cell response activation, clinical research is geared towards obtaining highly immunogenic antigens that can overcome immune evasion.

Moreover, lymphocytes that are capable of infiltrating the tumor to generate an antitumor immune response are also key players in the TME with clinical relevance. In fact, studies carried out in different tumors like ovarian cancer, have shown that the presence of CD3^+^ TILs [147] (or in which infiltrating and immunogenic properties are attributed to CD8^+^ TILs) is associated with a 5-year survival rate of 38%, compared to 4.5% in patients whose tumors do not have TILs. Their presence suggests the activation of antitumor responses to control tumor growth, which is also associated with a better response to chemotherapy [147]. Accordingly, lncPEP-derived neoantigens emerge as viable antitumoral molecules due to their immunogenic potential. Thus, these neoantigens provide a unique opportunity for developing personalized immunotherapy through the study of the T-cell receptors (TCR) of these TIL populations and their specific antigens [147,176], by harnessing their specific immunogenic properties to prime effective antitumor immune responses.

### 9.2. Low Mutational Burden Cancers Highlight an Unmet Need for Novel Neoantigens

Technological advances in NGS have made it possible to identify neoantigens derived from somatic mutations and use them in immunotherapy, especially in personalized vaccines [177]. The relevance of neoantigens in therapies such as the use of TILs is becoming increasingly evident, with many of the antigens associated with tumors being identified as T cell targets [177,178]. However, the studies in which these have shown promising results were conducted on tumors with a very high mutational burden, and it is unclear whether they will have the same effect on tumors with intermediate or low mutational burdens. Only a small percentage of mutations give rise to actual T cell epitopes, causing the type of tumor and its mutation rate to vary by up to five orders of magnitude. This difference in the number of somatic mutations has been shown to be related to cytotoxic T cell infiltration, such that the greater the number of mutant epitopes, the greater the number of TILs, which in turn is associated with longer patient survival [177,179].

Tumors with low mutational burdens, such as ovarian cancer, tend to show limited responses to current immunotherapies. Studies have shown that only 1.3% of mutations observed in HGSC are capable of eliciting a T cell response [180]. If we compare the predicted immunogenic mutations between HGSC and lung cancer, it is estimated that the number of MHC-I epitopes for each tumor type is 0.48 and 2.16 respectively, which translates into 12% and 51% of cases having a 90% probability of containing at least one neoantigen [177]. This means that therapy targeting these mutant neoantigens is very limited in tumors with low mutational burden, making it imperative to explore new sources of antigens, such as neo-Ags-lncRNA. Discovery of new neoantigens derived from lncRNAs, can help to overcome the limitations that exist in tumors with low mutational burden and in which immunotherapy was not achieving satisfactory results.

### 9.3. Immunological Safety and Off-Tumor Reactivity

Although neoAgs-lncRNAs are expected to show higher tumor specificity compared to mutation-derived antigens, safety concerns remain. Some lncRNAs display tissue-restricted but not tumor-exclusive expression, which may result in off-tumor toxicity if targeted therapeutically. This issue is particularly relevant for adoptive T cell therapies, where high-affinity receptors could cross-react with peptides expressed in healthy tissues.

In addition, the role of immune tolerance mechanisms is not fully understood. Low-level expression of lncRNA-derived peptides in normal tissues may induce tolerance, potentially limiting immunogenic responses. Comprehensive expression profiling across normal tissues and rigorous cross-reactivity testing will be essential before clinical implementation.

### 9.4. Clinical Translation and Regulatory Considerations

The identification and validation of lncPEPs as neoantigens could enable the design of personalized vaccines or adoptive therapy strategies targeting tumor-exclusive antigens, improving efficacy and reducing adverse effects associated with conventional therapies. However, there are several significant challenges to solve before applying these therapies in cancers that should be addressed.

On the one hand, tumor heterogeneity may mean that neoantigens and lncRNA derivatives show different expression patterns between patients, within the same tumor type, and even between different tumor regions, making it difficult to identify and validate them as immunogenic targets for vaccines or targeted therapies [181]. On the other hand, as mentioned before, tumors characterized by highly immunosuppressive tumor microenvironments, featuring low expression of MHC-I and other components of the antigen presentation machinery, may limit the immunogenicity of these peptides and reduce the efficacy of the T-cell response [182]. Therefore, patient stratification by cancer type and subtype would be necessary to identify immunotherapy responders.

Moreover, clinical translation of lncPEP-based therapies presents logistical and regulatory challenges. Personalized neoantigen strategies require complex workflows involving sequencing, antigen prediction, validation, and therapeutic manufacturing. Incorporating non-canonical peptides adds further complexity, as regulatory agencies require robust evidence supporting peptide origin, safety, and reproducibility. Standardization remains limited, particularly regarding analytical pipelines, validation criteria, and functional assays for non-canonical antigens. Regulatory frameworks developed for mutation-derived neoantigens may need adaptation to accommodate unconventional antigen sources. Large clinical studies will be necessary to demonstrate clinical benefit and feasibility of implementing these strategies at the bedside.

### 9.5. Technical and Computational Challenges

The identification of lncPEPs remains technically challenging due to their generally low abundance, transient expression, and incomplete annotation of lncRNA transcripts. The inclusion of non-canonical ORFs in proteogenomic analyses significantly expands the search space, increasing computational complexity and the risk of false-positive identifications. In addition, current peptide–HLA binding prediction tools are mainly trained using canonical peptides and frequent HLA alleles, which may reduce prediction accuracy for non-canonical sequences.

Another limitation is the uncertainty associated with translation evidence. Signals detected by ribosome profiling do not always correspond to stable peptide production, and functional validation remains necessary to confirm biological relevance. Progress in this field will require improved lncRNA annotation, larger immunopeptidomic datasets, and computational models specifically optimized for non-canonical antigen discovery.

### 9.6. Future Perspectives for lncPEP-Based Vaccines and Cell Therapies

Despite current limitations, lncPEPs represent a promising source of tumor antigens, particularly for tumors with low mutational burden. Their context-specific expression and potential recurrence across patient subsets support both personalized and semi-shared therapeutic approaches. Advances in mRNA vaccine platforms, nanoparticle delivery systems, and dendritic cell–based vaccines may facilitate the incorporation of lncPEP-derived epitopes into immunotherapeutic strategies.

Adoptive T cell therapies targeting lncRNA-derived antigens may also benefit from improvements in TCR engineering, safety mechanisms, and combination strategies with immune checkpoint inhibitors. Future studies integrating spatial transcriptomics, single-cell analyses, and functional genomics will help clarify the biological relevance of lncPEPs and support their translation into clinical applications.

## 10. Conclusions

lncPEPs are emerging as a previously underappreciated component of the tumor immunopeptidome, expanding the landscape of potential neoantigens beyond canonical protein-coding regions. Increasing evidence indicates that lncRNAs can encode small peptides capable of being processed and presented by HLA molecules and recognized by T cells, highlighting their relevance for cancer immunotherapy. Their context-specific expression and potential tumor selectivity make lncPEPs particularly attractive targets in malignancies with low mutational burden, where conventional mutation-derived neoantigens are limited. Advances in multi-omic technologies, including ribosome profiling, proteogenomics, and immunopeptidomics, have been essential for uncovering this non-canonical antigen repertoire and linking lncRNA biology to immune recognition mechanisms.

Despite these advances, important challenges remain regarding accurate identification, functional validation, safety assessment, and clinical translation of lncPEP-based targets. Continued integration of computational prediction, spatial and single-cell analyses, and functional genomics approaches will be necessary to prioritize clinically relevant candidates and understand their biological impact within the tumor microenvironment. Ultimately, lncRNA-derived neoantigens may contribute to the development of next-generation cancer vaccines, adoptive T-cell therapies, and biomarker-guided treatment strategies, offering new opportunities to improve immunotherapy outcomes in patients with limited therapeutic options.

## Figures and Tables

**Table 1:** Key differences between mutation-derived neoantigens and lncRNA-derived neoantigens.

Feature	Mutation-Derived Neoantigens	lncRNA-Derived Neoantigens
**Genomic origin**	Non-synonymous somatic mutations in coding regions	Non-coding transcripts containing smORFs
**Frequency**	Dependent on tumor mutational burden	Potentially abundant due to widespread ncRNA transcription
**Tumor specificity**	Often patient-specific	May include shared or recurrent antigens
**Detection approaches**	WES/WGS + prediction pipelines	RNA-seq, Ribo-seq, proteogenomics
**Expression patterns**	Linked to mutation presence	Often tissue- or context-specific
**Clinical relevance**	Widely explored in immunotherapy	Emerging and still under investigation
**Limitations**	Rare in low-mutational-burden tumors	Detection and validation challenges

## Data Availability

No new data were created or analyzed in this study. Data sharing is not applicable to this article.

## Abbreviations

APC	Antigen-presenting cell
BLI	Biolayer interferometry
CAR-T	Chimeric antigen receptor T cell
CTLA-4	Cytotoxic T-lymphocyte–associated protein 4
CTL	Cytotoxic T lymphocyte
DC	Dendritic cell
DDA	Data-dependent acquisition
DIA	Data-independent acquisition
DRiP	Defective ribosomal product
ELISA	Enzyme-linked immunosorbent assay
ELISpot	Enzyme-linked immunospot assay
EOC	Epithelial ovarian cancer
ER	Endoplasmic reticulum
ERAP	Endoplasmic reticulum aminopeptidase
ERK	Extracellular signal-regulated kinase
FACS	Fluorescence-activated cell sorting
FDR	False discovery rate
GTI-Seq	Global translation initiation sequencing
HGSOC	High-grade serous ovarian carcinoma
HLA	Human leukocyte antigen
IDH	Isocitrate dehydrogenase
IEDB	Immune Epitope Database
IFN-γ	Interferon gamma
IRES	Internal ribosome entry site
JAK	Janus kinase
KD	Dissociation constant
LC-MS/MS	Liquid chromatography–tandem mass spectrometry
lncPEP	Long non-coding RNA–derived peptide
lncRNA	Long non-coding RNA
m^5^C	5-methylcytidine
m^6^A	N6-methyladenosine
MAPK	Mitogen-activated protein kinase
MHC-I	Major histocompatibility complex class I
MHC-II	Major histocompatibility complex class II
MS	Mass spectrometry
mTOR	Mechanistic target of rapamycin
NF-κB	Nuclear factor kappa B
NGS	Next-generation sequencing
NOP	Neo-open reading frame peptide
PD-1	Programmed cell death protein 1
PD-L1	Programmed death-ligand 1
pHLA	Peptide–HLA complex
PI3K	Phosphatidylinositol 3-kinase
RBP	RNA-binding protein
Ribo-seq	Ribosome profiling sequencing
RNP	Ribonucleoprotein
RNA-seq	RNA sequencing
smORF	Small open reading frame
SPR	Surface plasmon resonance
STAT	Signal transducer and activator of transcription
TAP	Transporter associated with antigen processing
TCGA	The Cancer Genome Atlas
TCR	T cell receptor
TCR-T	T cell receptor–engineered T cell
TIL	Tumor-infiltrating lymphocyte
TME	Tumor microenvironment
VEGF	Vascular endothelial growth factor
WES	Whole-exome sequencing
WGS	Whole-genome sequencing
